# Microelements, Fatty Acid Profile, and Selected Biomarkers in Grass Carp (*Ctenopharyngodon idella*) Muscle Tissue: Seasonal Variations and Health Risk Assessment

**DOI:** 10.1007/s12011-024-04190-9

**Published:** 2024-05-09

**Authors:** Anton Kovacik, Marek Helczman, Julius Arvay, Marian Tomka, Marek Snirc, Ivona Janco, Martin Fik, Nikola Stefunkova, Rudolf Dupak, Lubos Harangozo, Katarina Tokarova, Eva Kovacikova, Tomas Jambor, Jaroslav Andreji, Peter Massanyi

**Affiliations:** 1 Institute of Applied Biology, Faculty of Biotechnology and Food Sciences, Slovak University of Agriculture in Nitra, Tr. A. Hlinku 2, 949 76 Nitra, Slovak Republic; 2 Institute of Food Sciences, Faculty of Biotechnology and Food Sciences, Slovak University of Agriculture in Nitra, Tr. A. Hlinku 2, 949 76 Nitra, Slovak Republic; 3 Institute of Biotechnology, Faculty of Biotechnology and Food Sciences, Slovak University of Agriculture in Nitra, Tr. A. Hlinku 2, 949 76 Nitra, Slovak Republic; 4 AgroBioTech Research Centre, Slovak University of Agriculture in Nitra, Tr. A. Hlinku 2, 949 76 Nitra, Slovak Republic; 5 Institute of Animal Husbandry, Faculty of Agrobiology and Food Resources, Slovak University of Agriculture in Nitra, Tr. A. Hlinku 2, 949 76 Nitra, Slovak Republic; 6 Reprofit International s.r.o., Hlinky 48/122, Pisarky, 603 00 Brno, Czech Republic

**Keywords:** Trace elements, Dorsal muscle, Biomarker, FAs, Biomonitoring, Risk assessment, Freshwater fish

## Abstract

The study assesses associations between microelement levels, fatty acid composition, and oxidative stress markers in grass carp muscle in the summer and autumn seasons. Additionally, various factors were considered, including the estimated daily intake (EDI), target hazard quotient (THQ), total THQ (TTHQ), and metal pollution index (MPI), to evaluate potential health risks for consumers. The microelements (Al, As, Ba, Cd, Co, Cr, Cu, Fe, Hg, Li, Mn, Ni, Pb, Se, Sr, and Zn) were determined using inductively coupled plasma optical emission spectrometry (ICP-OES), and total mercury was determined using cold-vapor atomic absorption spectroscopy (CV-AAS). Fatty acid profiling was realized using gas chromatography (GC) detection with a flame ionization detector (FID). The overall tendency of microelement levels was as follows: Fe > Zn > Al > Sr > Ba > Ni > Se > Cr> Cu > Mn > Pb > As > Li > Hg; <LOQ (below limit of quantification): Cd, and Co. The correlation analysis between concentrations of trace elements (Al, Ba, Cr, Cu, Mn, Li, Sr, Zn, and Hg) and the fatty acids (C16:0, C16:1, C18:1n9c, C18:2n6c, C20:4n6c, EPA, and DHA), as well as between Al, Ba, Fe, or Hg and oxidative stress markers (superoxide dismutase—SOD, glutathione peroxidase—GPx, or total antioxidant status—TAS), revealed statistically significant interactions in different seasons. THQ values were lower than 1; TTHQ values ranged from 0.27 to 0.76. The main toxic elements forming TTHQ were Hg and Ni (49%). The calculated health risk assessment indices indicate a low concentration of observed elements and low risks associated with the consumption of grass carp muscle from the tested location, although concentrations and THQ of mercury and nickel may be of slight concern.

**Supplementary Information:**

The online version contains supplementary material available at 10.1007/s12011-024-04190-9.

## Introduction

Fish meat remains a crucial source of fatty acids (FAs), particularly polyunsaturated omega-3 fatty acids, which are highly important in maintaining healthy cholesterol levels and preventing cardiovascular disease [1, 2]. The consumption of fish meat is popular [3], but along with it, question related to the benefits of such consumption due to the risks that threaten after the intake of environmental pollutants, such as mainly heavy metals and persistent organic pollutants (POPs) [4–12], which accumulate in aquatic animals. Several aquatic species widely used in nutrition, such as mussels, shrimps, or crabs, can pose health risks due to the high content of heavy metals in the tissues [13, 14]. Other aquatic organisms, such as microorganisms and plants, as well as fish can be used as indicators of water ecosystem pollution [15–18].

Microelements, such as aluminum—Al, arsenic—As, barium—Ba, cadmium—Cd, cobalt—Co, chromium—Cr, copper—Cu, iron—Fe, mercury—Hg, lithium—Li, manganese—Mn, nickel—Ni, lead—Pb, selenium—Se, strontium—Sr, and zinc—Zn, enter the environment through different pathways. It can be a natural pathway or an anthropological activity associated with mining, industry, agricultural activities, or urban pollution [19–21]. Blood biomarkers, oxidative stress markers, or fatty acids are some of the most useful bio-indicators for determining the effects of chemical stressors (e.g., heavy metals or POPs) in aquatic biota [6, 22–27]. Fish is a rich source of nutrition, particularly due to its high content of two omega-3 polyunsaturated fatty acids (PUFA): eicosapentaenoic acid (EPA) and docosahexaenoic acid (DHA). They have high structural diversity and bio-specificity, and they are very sensitive to stress conditions. FA biosynthesis is inhibited upon exposure to these pollutants, along with changes in FA desaturation and elongation processes. Long-term exposure to metals has been shown to likely alter the fatty acid composition of fish muscle through altered oxidative balance and reduced activity of certain mitochondrial enzymes [28–30]. It is important to note that not all trace elements are primarily toxic. Essential metals such as iron, zinc, which is required in the metabolism of proteins, nucleic acids, and lipids, or copper, of which the redox potential is utilized by several enzymes including mitochondrial cytochrome c oxidase, are essential in the nutrition of all organisms, and their deficiency may lead to serious negative health consequences [31, 32]. However, it is important that the intake of these micronutrients does not exceed the recommended daily allowance, as they also become toxic in excessive amounts. Nonessential microelements (e.g., As, Cd, Hg, or Pb), on the other hand, have no biological importance, so their intake into the body in any amount may pose a health risk [33, 34]. Biochemical and molecular biomarkers can be used as early warning signs of heavy metal contamination in organisms. Molecular biomarkers are the first to respond to heavy metal contamination, followed by biochemical and physiological response levels [35–37]. Superoxide dismutase (SOD) or glutathione peroxidase (GPx) are very potent contamination indicators of heavy metals such as iron (Fe) or mercury (Hg). Malondialdehyde (MDA) is also an important bio-indicator of heavy metal pollution. Accumulation of heavy metals in fish flesh may be one of the reasons responsible for the increase in lipid peroxidation [38–41].

Consequently, the aim of this study was to evaluate whether the microelements content could affect the fatty acid profiling, body weight, or total length, and oxidative status markers (SOD, GPx, MDA, and TAS) in grass carp (*Ctenopharyngodon idella*) muscle. Potential health risks for consumers were evaluated using several instruments (the target hazard quotient—THQ, total target hazard quotient—TTHQ, and metal pollution index—MPI). Currently, there is a lack of information about fatty acid composition and trace element accumulation in grass carp muscle from this locality (an area with intensive agricultural activity lasting decades, connection with wastewater treatment plant), as well as the extent of human exposure and potential health consequences. Hence, there is a need for detailed information to assess the health risks for consumers of fish in general.

## Material and Methods

### Study Area and Sampling

Fish were collected from a university experimental pond (Kolinany, Slovak Republic; 48° 21′ 14.6″ N 18° 13′ 03.2″ E) between June and November. The pond, subject to strong agricultural activity and connected to a wastewater treatment plant, serves dual purposes for commercial fish breeding and sport fishing. Despite its multiple uses, no previous studies have assessed the potential accumulation of toxic elements in fish muscle tissue at this location.

Fish were caught by a seine net and immediately transported to the laboratory [42]. A total of 36 grass carp (*Ctenopharyngodon idella*) were collected. The individuals were categorized into two groups (*n* = 18 each) based on the season of capture: summer season (June to August), and autumn season (September to November).

During the slaughter process, an authorized person handled the fish, strictly adhering to both European (Directive 2010/63/EU) [43] and national regulations, and the Slovak University of Agriculture in Nitra’s Ethics Committee for the Protection of Animals Used for Scientific and Teaching Purposes statute. After slaughtering (cranial concussion and following decapitation), the dorsal muscle without skin and bones were stored at − 20 °C until further analyses at the Institute of Applied Biology. Standard morphometric (body weight—BW and standard length—SL) data was previously published (SL was 431.7 ± 38.9 mm for summer season, 414.7 ± 24.5 mm for autumn season; BW was 1383 ± 364.9 g for summer season, 1243.5 ± 244.0 g for autumn season) [42]. Total length (TL) of fish was 510.0 ± 45.2 mm for summer season and 491.9 ± 24.4 mm for autumn season.

### Sample Pre-preparation for ICP-OES

Using the high-performance microwave digestion machine Ethos UP (Milestone Srl, Sorisole, BG, Italy), dorsal muscle (up to 0.5 g) was prepared for ICP OES analysis using a closed-vessel microwave acid digestion method (solution of 5 mL HNO_3_ and 1 mL H_2_O_2_) [25]. To reduce contamination, all the chemicals utilized in this technique had high purity grades (designed for trace analyses).

### Detection of Microelements (ICP-OES)

The concentration of microelements (Al, As, Ba, Cd, Co, Cr, Cu, Fe, Li, Mn, Ni, Pb, Se, Sr, and Zn) was analyzed by Inductively Coupled Plasma Optical Emission Spectrometry (ICP-OES) using the ICP OES 720 (Agilent Technologies, Santa Clara, CA, USA) [44]. ICP-OES measurement parameters were follows: RF power 1.30 kW; plasma flow 15.0 L/min; auxiliary flow 1.50 L/min; nebulizer flow 0.85 L/min; replicated read time 5.00 s; instrument stabilization 15 s; sample uptake delay 25 s; pump rate 15 rpm; rinse time 10 s; element wavelengths (nm): Al 328.068, As 188.980, Ba 455.403, Cd 226.502, Co 228.615, Cr 267.716, Cu 324.754, Fe 234.350, Li 670.783, Mn 257.610, Ni 231.604, Pb 220.353, Se 196.026, Sr 407.771, and Zn 206.200. Detection limits (μg/L) of measured trace elements were follows: Al 0.20, As 1.50, Ba 0.03, Cd 0.05, Co 0.20, Cr 0.15, Cu 0.30, Fe 0.10, Li 0.06, Mn 0.03, Ni 0.30, Pb 0.80, Se 2.00, Sr 0.01, and Zn 0.20. Quantification limits (μg/L) of measured trace elements were follows: Al 0.66, As 4.95, Ba 0.10, Cd 0.17, Co 0.66, Cr 0.50, Cu 0.99, Fe 0.33, Li 0.20, Mn 0.10, Ni 0.99, Pb 2.64, Se 6.60, Sr 0.03, and Zn 0.66. Certified reference materials (ERM CE278K, ERM-BB184, and BCR-274, Sigma-Aldrich Production GmbH, Switzerland) and multielement standard solution 5 for ICP (Sigma-Aldrich Production GmbH, Switzerland) were used for verification and control in all analyses. The recoveries were 93% for Al, 98% for As, 89% for Ba, 98% for Cd, 99% for Co, 94% for Cr, 91% for Cu, 99% for Fe, 93% for Li, 99% for Mn, 98% for Ni, 99% for Pb, 93% for Se, 91% for Sr, and 93% for Zn. The concentrations of elements were expressed as milligram per kilogram wet weight (mg/kg).

### Detection of Mercury (CV-AAS)

For the detection of total mercury concentration (Hg), a selective mercury analyzer (AMA-254; Altec, Prague, Czech Republic) based on Cold Vapor Atomic Absorption Spectrometry (CV-AAS) was employed. The defrosted dorsal muscle samples were directly measured, with a mercury detection limit of 1.5 ng/kg.

### Sample Pre-preparation for Chromatographic Analysis

Lipids from dried dorsal muscle were extracted by modified methods, according to Bligh and Dyer [45] and Folch et al. [46]. Muscle lipids were esterified into fatty acid methyl esters (FAME) by mixing 0.2 g of the sample with 7.6 mL of a chloroform-methanol-deionized water solution (2:4:1.6 v/v/v) at 250 rpm for three hours in a 15 mL plastic vial (Unimax 2010, Heidolph, Germany). Following a 2-min centrifugation at 2795×*g* (Rotina 420, Hettich, Germany), the samples were promptly filtered via a SPE vacuum manifold (Agilent Technologies, USA). Then, 1 mL of chloroform, 1 mL of KCl (2 mol/L), and 0.9 mL of deionized water were added to each sample. After that, the samples were centrifuged for 5 min at 1006×*g* using a Rotina 420, Hettich, Germany. Next, anhydrous sodium sulfate and a SPE vacuum manifold (Agilent Technologies, USA) were used to recover and dry the extract's bottom phase. 1.45 mL of extract was transferred to a microtube for methylation. Next, 500 μL of hexane and 50 μL of KOH (methanolic, 2 mol/L) were added, and the mixture was vortexed using a vortex stirrer (Heidolph, Germany). Finally, before analysis, 1 mL of the FAME extract was moved to a GC vial.

### Fatty Acid Profiling—Separation and Quantification

Gas chromatography (GC) detection using a flame ionization detector (FID) on an Agilent 7890A (Agilent Technologies, USA) was used to quantify fatty acids. Using the CombiPAL autosampler, 1 μL of the sample was introduced into the device. Fatty acid methyl esters were separated using an Agilent Technologies, USA, HP-88 (60 m × 0.25 mm × 0.20 μm). The data was processed online using the Agilent OpenLab ChemStation. Instrument calibration was performed using a Supelco 37-component FAME Mix (TraceCERT, Supelco, USA). We used an Agilent 7890B gas chromatograph (Agilent Technologies, USA) with an Agilent 5977A mass detector (Agilent Technologies, USA) to determine the qualitative composition of the samples.

### Sample Pre-preparation for Biomarker Measurements

Samples for biomarker evaluation were prepared using a modified method according to Tvrdá et al. [47]. Dissected muscle tissue was washed with chilled phosphate buffer saline (PBS; Sigma-Aldrich, USA) and cut into smaller pieces while keeping it on ice. Tissue was transferred to a homogenizer, and RIPA buffer with protease inhibitor was added (Sigma-Aldrich, USA) (500 μL RIPA buffer for every 10 mg of tissue). The sample was sonicated for 2 min at a power of about 180 watts (in rounds of 10 seconds sonication/10 s rest for each cycle). Throughout the sonication, the sample was maintained on ice. The cell debris was then pelleted by centrifuging at 10,822 × *g* for 20 min at 4 °C. The supernatant was then transferred to a new microfuge tube without disturbing the pellet.

### Measurements of Biomarkers

The obtained supernatant was used for the analysis of selected biomarkers. Superoxide dismutase (SOD), glutathione peroxidase (GPx), total antioxidant status (TAS), and total protein (TP) activity was measured using the Randox commercial kits (Randox Laboratories, Crumlin, UK) and the automated analyzer Randox RX Monaco (Randox Laboratories, Crumlin, UK) according to standard methodology. MDA concentrations were detected with the help of the TBARS assay according to Tvrdá et al. [48]. Every sample underwent treatment involving 5% sodium dodecyl sulfate and 0.53% thiobarbituric acid (TBA) dissolved in 20% acetic acid, which was then modified to a pH of 3.5 using NaOH. After that were boiled at a temperature range of 90–100 °C for 1 h. After boiling, the samples were cooled on ice for 10 min and then centrifuged at 1750×*g* for another 10 min. Supernatant was then utilized to detect the formation of products from the MDA-TBA reaction. The supernatant measured at 530–540 nm was used to analyze the reaction products of MDA and TBA, which are formed under high temperature and acidic conditions. The results (SOD, GPx, TAS, and MDA) were converted and expressed as units per milligram of total protein (SOD), units per gram of total protein (GPx), and micromoles per gram of total protein (MDA and TAS).

### Risk Assessment and Index Calculations

Fulton’s Condition Factor (*K*) is a useful tool in fisheries science, providing a simple yet powerful metric for assessing the health and condition of fish populations, guiding management decisions, and contributing to the conservation of aquatic ecosystems. K serves as an effective indicator of the overall health and condition of individual fish and fish populations [49, 50]. This factor is calculated by dividing the weight of a fish by its length with the result then standardized against a reference condition [50]:

$$K=\frac{W}{L^3}\times 100$$

where *W* is the total weight of fish (g), *L* is the total length of fish (cm).

Using estimated daily intake (EDI), we assess the amount of a particular substance that an individual might be exposed daily from various sources (e.g., fish meat). EDI values were used for target hazard quotient calculation. The EDI was calculated using the following equation [51, 52]:

$$\mathrm{EDI}=C\times \mathrm{IR}/\mathrm{BW}$$

where EDI is the estimated daily intake (mg/kg/body weight/day), *C* is the mean concentration of microelement in muscle (mg/g wet weight), IR is the daily ingestion rate (g/person/day), and BW is the mean body weight (kg). The average body weight of an adult in Europe has been set at 70 kg [53], and the daily consumption of fish meat is 65.75 g/day [54].

Target hazard quotient (THQ) is the estimated health risk of consumers owing to heavy metal contaminated fish consumption using an oral reference dose. THQ < 1 indicates that consumption of such fish has a health advantage and that consumers are safe; however, THQ > 1 indicates that there are significant adverse health risks. The THQ was calculated using the following equation [55]:

$$\mathrm{THQ}=\frac{\mathrm{EDI}}{\mathrm{RfD}}$$

where EDI is the estimated daily intake (mg/kg/day), RfD is the oral reference dose (mg/kg/day). Oral reference dose according to US EPA [56] is Al = 1.0 mg/kg/day, Ba = 0.20 mg/kg/day, Cr = 1.50 mg/kg/day, Cu = 0.04 mg/kg/day, Fe = 0.70 mg/kg/day, Hg = 0.0003 mg/kg/day, Li = 0.002 mg/kg/day, Mn = 0.14 mg/kg/day, Ni = 0.02 mg/kg/day, Sr = 0.6 mg/kg/day, Zn = 0.3 mg/kg/day.

TTHQ, total target hazard quotient, was calculated by the sum of the hazard quotients of all metals [57].

$$\sum \mathrm{THQ}\left(\mathrm{TTHQ}\right)=\mathrm{THQ}\left(\mathrm{Al}\right)+\mathrm{THQ}\ \left(\mathrm{Ba}\right)+\mathrm{THQ}\left(\mathrm{Cr}\right)+\mathrm{THQ}\left(\mathrm{Cu}\right)+\mathrm{THQ}\left(\mathrm{Fe}\right)+\mathrm{THQ}\left(\mathrm{Hg}\right)+\mathrm{THQ}\left(\mathrm{Li}\right)+\mathrm{THQ}\left(\mathrm{Mn}\right)+\mathrm{THQ}\left(\mathrm{Ni}\right)+\mathrm{THQ}\left(\mathrm{Sr}\right)+\mathrm{THQ}\left(\mathrm{Zn}\right)$$

Metal pollution index (MPI) is a quantitative instrument for assessing and expressing the level of metal pollution in each environment. The MPI was calculated using the following equation [58]:

$$\mathrm{M}\mathrm{PI}={\left(\mathrm{M}1\times \mathrm{M}2\times \mathrm{M}3\times \dots \mathrm{M}\mathrm{n}\right)}^{1/n}$$

where M1 is the concentration of first metal, M2 is the concentration of second metal, M3 is the concentration of third metal, M*n* is the concentration of “*n*” metal (mg/kg wet weight) in a certain tissue.

### Statistical Analyses

The results are presented as mean ± standard deviation. Basic statistics and Kolmogorov-Smirnov normality tests were performed. To evaluate the differences between the monitored experimental groups, we used unpaired *t*-test. In case of not passing the normality test, the nonparametric Mann-Whitney test was used to evaluate the differences between the groups (GraphPad Prism 8.0.1.; GraphPad Software Incorporated, San Diego, California, USA). Spearman correlation analysis was used to evaluate the associations between microelements and monitored biomarkers (STATGRAPHICS Centurion, ©StatPoint Technologies, Inc., USA). Hierarchical clustering on principal components analysis (HCPC) was performed on the dataset to identify groups of samples according to the variable season. Principal component analysis (PCA) was used to reduce the dimensionality of data by extracting continuous variables encompassing the most significant information. PCA and HCPC analyses were performed in RStudio program using the “FactoMineR” and “factoextra” packages [59] with the RStudio software, version 2023.09.1 [60] and MS Excel with the XLSTAT package [61].

## Results

**Fig. 1:**
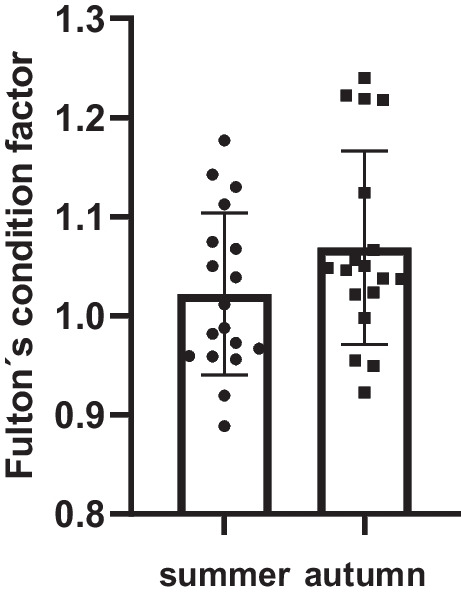
Calculated Fulton’s condition factor (*K*) for grass carp (each column represent mean ± SD; scatter plot represents each calculated value)

The calculated values of Fulton’s condition factor (*K*) are presented in Fig. 1. Almost all individuals evaluated had *K* values between 0.8 and 1.2, indicating that they were in optimal health. The highest *K* values (from 1.22 to 1.24) were observed for four individuals in autumn group, suggesting very slight overweight. The lowest calculated value was 0.88 (summer group), indicating that none of the studied individuals were malnourished. No significant differences in *K* values were observed between the monitored seasons.

The levels of trace elements across the monitored seasons (summer and autumn) are presented in Table 1. The highest levels were recorded for Fe (15.32–57.42 mg/kg). The lowest levels were recorded for Hg (0.012–0.053 mg/kg). The overall tendency of microelement levels was as follows: Fe > Zn > Al > Sr > Ba > Ni > Se > Cr > Cu > Mn > Pb > As > Li > Hg; < LOQ (below limit of quantification): Cd, and Co. The concentration of Al was significantly different between observed seasons, with the higher level observed in autumn (*p* < 0.0001). The same tendency was observed for Cr (*p* < 0.0001), Fe (*p* < 0.0001), and Ni (*p* < 0.0002). A decreasing trend in the concentration of elements in autumn season was recorded for Ba (*p* < 0.05), Li (*p* < 0.0001), and Sr (*p* < 0.01). Hg, Mn, and Zn levels were higher in summer season compared to autumn, although statistical significance was not observed. Elements such as Cd and Co, were lower than the LOQ in both Seasons. Levels of As were lower than the LOQ in more than 88% of the samples, levels of Pb were lower than the LOQ in more than 66% of the samples, and levels of Se were lower than the LOQ in almost 50% of the samples. Therefore, we excluded these three elements from further statistical analyses and index calculations.

**Table 1:** Microelements concentrations (mg/kg wet weight) in grass carp dorsal muscle among seasons

Season/microelement	Summer	CV%	Autumn	CV%	*p* value
Al	17.38 ± 4.95	28.50	28.92 ± 5.63	19.46	**0.0001**
As	0.16 ± 0.34^14<LOQ^	-	< LOQ	-	-
Ba	5.94 ± 1.08	18.25	5.59 ± 0.98	17.52	**0.0239**
Cr	0.75 ± 0.15	19.65	1.57 ± 0.71	44.89	**0.0001**
Cu	1.07 ± 0.31	28.63	1.11 ± 0.16	14.51	0.1916
Fe	21.10 ± 4.99	23.68	36.99 ± 9.35	25.28	**0.0001**
Hg	0.03 ± 0.01	40.45	0.02 ± 0.01	22.40	0.0613
Li	0.06 ± 0.05	81.96	0.02 ± 0.01	76.40	**0.0001**
Mn	0.81 ± 0.13	16.51	0.78 ± 0.26	33.07	0.0790
Ni	2.13 ± 1.24	58.05	4.29 ± 1.85	43.02	**0.0002**
Pb	0.06 ± 0.13^14<LOQ^	-	0.21 ± 0.45^10<LOQ^	-	-
Se	1.36 ± 1.92^9<LOQ^	-	1.06 ± 1.40^8<LOQ^	-	-
Sr	10.02 ± 1.45	14.50	8.64 ± 2.41	27.89	**0.0064**
Zn	28.17 ± 6.59	23.38	26.41 ± 4.47	16.93	0.3549

^1^Results are presented as Mean ± SD; *CV%*, coefficient of variation; ^(n<LOQ)^ indicates how many samples were below LOQ; Cd and Co were below LOQ in both seasons; bold *p*-values are significantly different

Representative report of Fatty acid profiling is shown in Supplementary material (Figure S1). Composition of fatty acids (% of total FAs) of grass carp muscle tissue is presented in Table 2. In determining the total fatty acid ratio, we were able to detect the following fatty acids: C16:0; C16:1; C17:0; C18:0; C18:1n9c; C18:2n6c; C20:4n6c; EPA and DHA. In the studied fish meat samples, the significantly higher (*p* < 0.05) proportion of C18:1n9c (oleic acid) was detected in summer season (35.30 ± 13.90%) than in autumn season (25.68 ± 8.21%). C16:0 (palmitic acid) constituted the second largest proportion in the samples, with the higher values detected in autumn season, 25.25 ± 3.22%, vs. autumn season, 20.70 ± 4.44%, demonstrating a statistical difference (*p* < 0.01) between seasons. On contrary, the lowest proportion of fatty acids in the samples consisted of C16:1 (palmitoleic acid), undetectable in the samples from summer season, and in autumn season, its proportion in samples was 7.94 ± 3.41%. Stearic acid (C18:0) was detected at 14.39 ± 2.54% in summer season and 12.27 ± 2.1% in autumn season, suggesting a statistical difference (*p* < 0.01). Another statistically significant difference (*p* < 0.001) was observed between seasons for linoleic acid (C18:2n6c) where a proportion of 4.92 ± 1.67% was detected in summer and 6.94 ± 1.05% in autumn. EPA (eicosapentaenoic acid) and DHA (docosahexaenoic acid) had the statistically significant (*p* < 0.05) higher proportion in summer season.

**Table 2:** Composition of fatty acids (% of total FAs in lyophilised fish sample) in grass carp among seasons

Fatty acids (%)	Summer	CV%	Autumn	CV%	*p* value
C16:0	20.70 ± 4.44	21.47	25.25 ± 3.22	12.79	**0.0012**
C16:1	*nd*	-	7.94 ± 3.41	42.95	-
C17:0	5.39 ± 1.60	29.61	5.48 ± 2.51	45.90	0.9049
C18:0	14.39 ± 2.54	17.67	12.27 ± 2.11	17.25	**0.0099**
C18:1n9c	35.30 ± 13.90	39.40	25.68 ± 8.21	31.96	**0.0163**
C18:2n6c	4.92 ± 1.67	33.97	6.94 ± 1.05	15.09	**0.0001**
C20:4n6c	6.83 ± 1.95	28.60	6.86 ± 1.54	22.49	0.9499
EPA	5.15 ± 2.28	44.34	3.72 ± 1.42	38.15	**0.0312**
DHA	7.32 ± 1.79	24.46	5.86 ± 1.78	30.41	**0.0201**
Total SFA	40.49 ± 8.22	20.31	42.99 ± 6.40	14.90	0.3152
Total MUFA	35.30 ± 13.90	39.40	33.62 ± 8.77	26.09	0.6678
Total PUFA	24.21 ± 6.20	25.60	23.38 ± 4.48	19.16	0.6508
Total n-3	12.46 ± 3.84	30.79	9.58 ± 3.07	32.08	**0.0182**
Total n-6	11.75 ± 3.03	25.81	13.80 ± 1 84	13.34	**0.0194**
n-6/n-3	1.02 ± 0.37	36.68	1.53 ± 0.38	24.63	**0.0002**

^1^Results are presented as mean ± SD; *CV%*, coefficient of variation; bold *p*-values are significantly different; *nd*, not detected; *EPA*, eicosapentaenoic acid; *DHA*, docosahexaenoic acid; *SFA*, saturated fatty acids; *MUFA*, monounsaturated fatty acids; *PUFA*, polyunsaturated fatty acids, n-3 = EPA + DHA, n-6 = C18:2n6c + C20:4n6c, n6/n3 ratio

Total SFA (saturated fatty acid), total MUFA (mono-unsaturated fatty acid), total PUFA (poly-unsaturated fatty acid), total n-3, total n-6, and n6/n3 ratio are also presented in Table 2. The highest proportion was found for SFA, with the higher percentage during autumn season. MUFA had the second highest representation in the samples, with the higher values detected in summer season. PUFAs had the lowest proportion, with the higher percentage found in summer season. No statistically significant difference was observed in either case between the different seasons. Statistically significant difference (*p* < 0.05) was observed between seasons for total n-3 fatty acids and total n-6 fatty acids. For n-6/n-3 ratio, there was statistically significant difference between seasons (*p* < 0.001) with higher ratio in autumn season.

Table 3 shows the concentrations of total proteins, triacylglycerol, SOD, GPx, MDA, and TAS for fish muscle lysate. The total protein content was primarily used for conversion of oxidative stress biomarker results. The concentration of GPx was significantly higher in summer season (*p* < 0.001) than in autumn. Higher values of SOD and MDA were recorded in autumn season, but without statistical significance. No significant differences were found for TAS between seasons.

**Table 3:** Selected biomarkers analyzed in fish muscle lysates in different seasons

Biomarkers	Summer	CV%	Autumn	CV%	*p* value
TP (g/L)	5.14 ± 1.59	30.93	6.69 ± 2.10	31.41	**0.0174**
SOD (U/mg TP)	2.93 ± 1.95	66.82	3.48 ± 1.53	44.00	0.3538
GPx (U/g TP)	74.26 ± 31.88	42.93	38.27 ± 40.01	104.54	**0.0052**
MDA (μmol/g TP)	0.67 ± 0.42	63.14	0.89 ± 0.52	58.00	0.1569
TAS (μmol/g TP)	93.59 ± 23.65	25.26	82.54 ± 26.99	32.71	0.1998

^1^Results are presented as mean ± SD. *CV%*, coefficient of variation; bold *p*-values are significantly different; *TP*, total proteins; *SOD*, superoxide dismutase; *GPx*, glutathione peroxidase; *MDA*, malondialdehyde; *TAS*, total antioxidant status

Results of Spearman correlation analysis (statistically significant correlations) in different seasons are presented in Table 4. In summer season, statistically significant positive correlations were found between Cr concentration and TL, W, C16:0, C18:0, C18:2n6c, and SFA. Statistically significant positive correlations were also found between Cu concentration and C16:0, C18:2n6c, SFA and total n-6 fatty acids. Concentrations of Li were found to positively correlate with C16:0 and the total n-6 fatty acids. Mn concentration was found to positively correlate with C16:0, C18:0, and SFA. In this season, a group of elements (Cu, Hg, and Zn) was negatively correlated with C18:1n9c and with MUFA. Zn was also positively associated with C20:4n6c, EPA, DHA, and total n-3 fatty acids. Al and Ba were positively associated with oxidative status markers (SOD, GPx, or TAS).

**Table 4:** Statistically significant correlations (Spearman) between concentrations of microelement in the fish muscle and selected biomarkers in different seasons

Summer season				Autumn season			
Element	Marker	*R*	*p* value	Element	Marker	*R*	*p* value
Cr	TL	0.5611	0.0207	Al	TL	0.5137	0.0342
Cr	W	0.4985	0.0399	Cu	TL	− 0.4938	0.0418
Cr	C16:0	0.4799	0.0479	Fe	TL	− 0.6139	0.0114
Cu	C16:0	0.6450	0.0078	Sr	TL	0.6056	0.0125
Li	C16:0	0.5253	0.0303	Fe	W	− 0.5542	0.0223
Mn	C16:0	0.4819	0.0469	Sr	W	0.4799	0.0479
Cr	C18:0	0.4881	0.0442	Li	C16:0	− 0.5459	0.0244
Mn	C18:0	0.5604	0.0209	Al	C16:1	0.8550	0.0009
Hg	C18:1n9c	− 0.4799	0.0479	Ba	C16:1	0.5588	0.0304
Cu	C18:1n9c	− 0.5129	0.0345	Al	C18:2n6c	0.7434	0.0022
Zn	C18:1n9c	− 0.5212	0.0317	Ba	C18:2n6c	0.5150	0.0337
Al	C18:2n6c	− 0.5542	0.0223	Li	EPA	0.5624	0.0204
Cr	C18:2n6c	0.5356	0.0272	Li	Total n-3	0.4985	0.0399
Cu	C18:2n6c	0.5604	0.0209	Li	n-6/n-3	− 0.5108	0.0352
Zn	C20:4n6c	0.5604	0.0209	Hg	GPx	0.5273	0.0297
Hg	EPA	0.4925	0.0423	Fe	GPx	− 0.5150	0.0337
Zn	EPA	0.5256	0.0302	Hg	TAS	0.6285	0.0096
Zn	DHA	0.5005	0.0390				
Cr	SFA	0.4799	0.0479				
Cu	SFA	0.5418	0.0255				
Mn	SFA	0.5088	0.0359				
Hg	MUFA	− 0.4799	0.0479				
Cu	MUFA	− 0.5129	0.0345				
Zn	MUFA	− 0.5212	0.0317				
Zn	Total n-3	0.4840	0.0460				
Cu	Total n-6	0.6842	0.0048				
Li	Total n-6	0.5150	0.0337				
Ni	Total n-6	0.5046	0.0375				
Al	n-6/n-3	− 0.7461	0.0021				
Al	SOD	0.5088	0.0359				
Ba	GPx	0.6140	0.0114				
Ba	TAS	0.5253	0.0303				

In autumn season, a group of elements (Al, Cu, Fe, and Sr) was significantly correlated with TL or body weight of fish. In this season, statistically significant negative correlations were found between Li concentration and C16:0 and n-6/n-3 ratio. Al and BA were positively associated with C16:1 and C18:2n6c. Li was also positively correlated with the EPA and total n-3 fatty acids. Hg was positively correlated with GPx and TAS. In this season, a statistically significant negative correlation between Fe and GPx was also found.

**Fig. 2:**
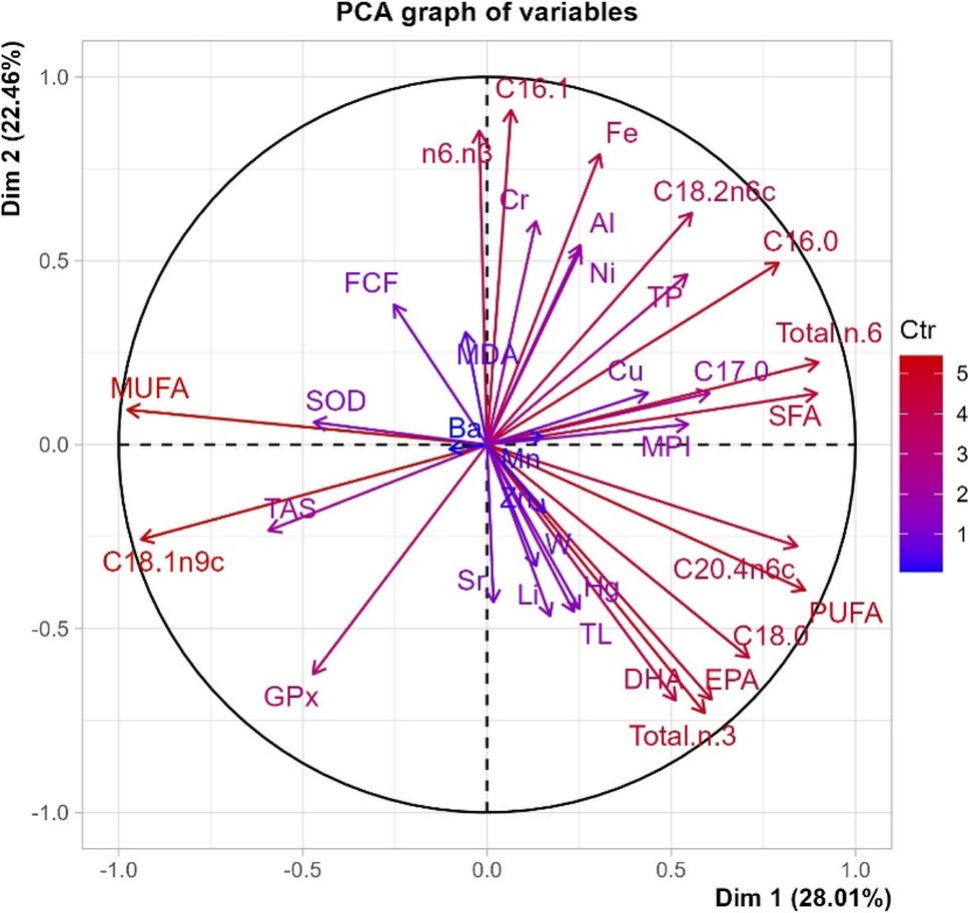
Correlation circle of variables with the contribution of each variable

The principal component analysis (PCA) revealed that 50.47% of the total variation could be effectively explained by the first two principal components (PCs) (Fig. 2), with eigenvalues of 9.80 and 7.86, respectively. The season was used as a quantitative variable to illustrate the distance between individuals according to The Wilks test (*p* < 0.05).

PC1, accounting for 28.01% of the inertia, contrasted fatty acid contents Total.n.6 (loading value 0.900), SFA (loading value 0.895), PUFA (loading value 0.863), C20.4n6c (loading value 0.842), C16:0 (loading value 0.792), and C18:0 (loading value 0.711, with MUFA (loading value − 0.976), and C18:1n9c (loading value − 0.939).

PC2, explaining 22.46% of the inertia, clearly reflected the different values for C16:1 (loading value 0.91) and n6/n3 (loading value 0.853), and Fe concentration (loading value 0.79), contrasting with total n-3 (loading value − 0.73), DHA (loading value − 0.695), and EPA (loading value − 0.692).

**Fig. 3:**
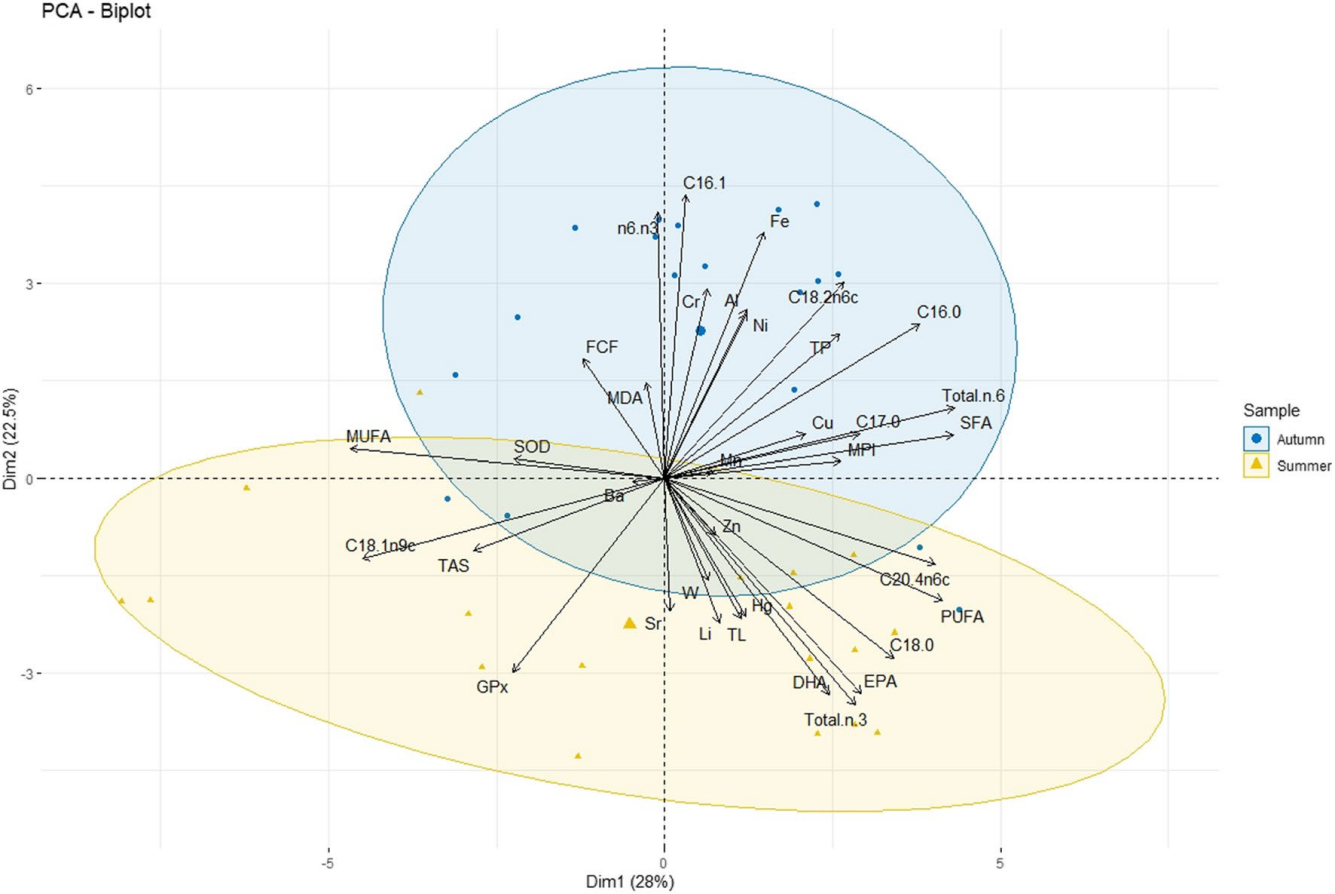
Map of Dim 1 and Dim 2 with observations (centroids and 95% confidence eclipses)

Figure 3 shows the confidence ellipses of individuals. Dimension 1 opposes individuals on the right side of the graph, characterized by strongly positive coordinates on the *x*-axis from summer season (S-7, S-6, S-2, S-17, and S-16) and individual A-1 from autumn season, to individuals on the left side of the graph, such as S-8, S-11, A-7, A-6, A-9, A-4, S-10, and S-9, characterized by strongly negative coordinates on the *x*-axis. The group of individuals S-7, S-6, A-1, S-2, S-17, and S-16 is characterized by high values for variables like C18:0, Total n-3, PUFA, DHA, EPA, C20:4n6c, W, TL, Hg, and Total n-6 and simultaneously low values for the variables MUFA, C16:1, n6/n3, and C18:1n9c. The group in which the individuals S-8, S-11, A-4, S-10, and S-9 stand shares high values for the variables C18:1n9c, MUFA, TAS, and GPx, and simultaneously low values for the variables like SFA, Total n-6, C16:0, C17:0, C20:4n6c, PUFA, Cr, C18:2n6c, C18:0, and MPI. The group in which the individuals A-7, A-6, and A-9 stand characterized by a negative coordinate on the axis is sharing high values for the variables C16:1, Fe, Cr, C16:0, C18:2n6c, TP, n6/n3, Ni, Al, and C17:0, and low values for the variables GPx, Li, Sr, DHA, and TAS.

The dimension 2 opposes individuals at the top of the graph, characterized by strongly positive coordinates on the *y*-axis (A-7, A-6, and A-9), contrast with individuals at the bottom of the graph, characterized by strongly negative coordinates on the y-axis (S-8, S-11, A-4, S-10, and S-9). The group containing individuals A-7, A-6, and A-9 exhibits high values for the variables C16.1, Fe, Cr, C16:0, C18:2n6c, TP, n6/n3, Ni, Al, and C17:0, while simultaneously displaying low values for the variables GPx, Li, Sr, DHA, and TAS. On the other hand, the group with individuals S-8, S-11, A-4, S-10, and S-9 shares high values for the variables C18:1n9c, MUFA, TAS and GPx, while simultaneously having low values for the variables SFA, Total n-6, C16:0, C17:0, C20:4n6c, PUFA, Cr, C18:.2n6c, C18:0, and MPI.

The main purpose of applying hierarchical clustering on principal components analysis (HCPC) is to extract hidden patterns in the dataset and present them visually for enhanced comprehension. Initially, PCA analysis was performed, and the eigenvalues obtained through PCA were used to determine the number of principal components (PCs) to retain, ensuring that the most significant features were retained while trivial ones were ignored. The first ten PCs were selected for HCPC analysis, resulting in the classification of individuals into three clusters, as displayed in the cluster dendrogram and factor map in Supplementary material (Figure S2).

- Cluster 1: This cluster comprises individuals belonging to summer seasons (S-8, S-9, S-10, S-11, S-12, S-14, S-15, and S-18), along with two individuals from autumn season (A-3, A-4). Characterized by high values for the variables C18:1n9c, MUFA, TAS, GPx, and SOD, this group simultaneously exhibits low values for the variables Total n-6, C16:0, SFA, C18:2n6c, TP, PUFA, C20:4n6c, Cr, Fe, and C17:0.
- Cluster 2: Comprising individuals exclusively from autumn season (such as A-7, A-8, A-9, A-10, A-11, A-12, A-14, and A-15), this cluster is characterized by high values for the variables C16:1, Fe, Cr, n6/n3, Al, C16:0, Ni, C18:2n6c, TP, and FCF. Simultaneously, it shows low values for the variables GPx, C18:0, Li, Total n-3, DHA, EPA, Sr, C18:1n9c, and TL.
- Cluster 3: This cluster includes the remaining individuals from summer season (such as S-6, S-7, S-16, and S-17) and some individuals from autumn season (A-1 and A-2). Characterized by high values for variables C18:0, PUFA, Total n-3, EPA, DHA, C20:4n6c, Li, SFA, Total n-6, and Hg, this group simultaneously exhibits low values for the variables MUFA, C16:1, n6/n3, SOD, C18:1n9c, Fe, and Ni.

**Fig. 4:**
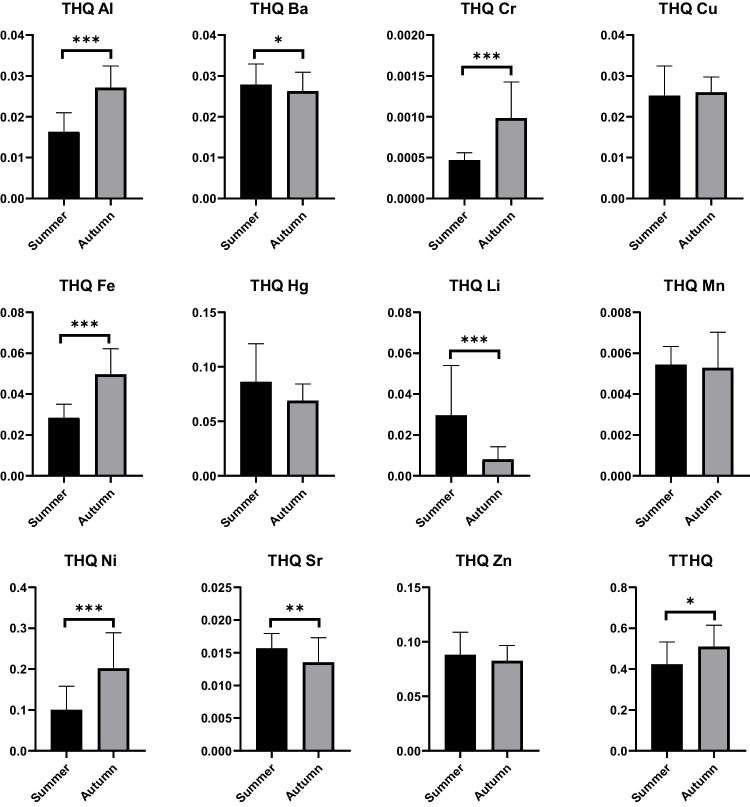
Results of target hazard quotient (THQ) and total target hazard quotient (TTHQ), which represent the sum of the target hazard quotients of all elements in grass carp muscle samples; columns represent mean ± SD. The level of significance was set at *p < 0.05; **p < 0.01; ***p < 0.001

**Fig. 5:**
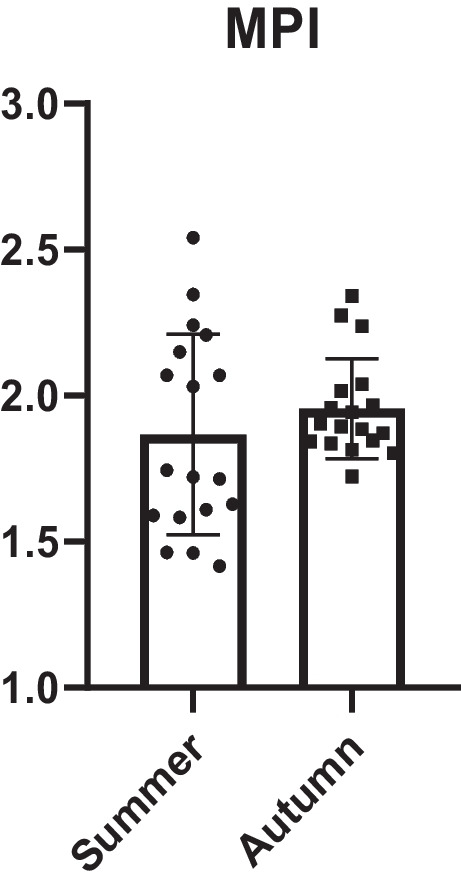
Results of metal pollution index (MPI) for observed microelements calculated based on recommended daily intake of all elements in grass carp muscle samples; columns represent mean ± SD; scatter plot represents each calculated value

The target hazardous quotients (THQ), total target hazardous quotient (TTHQ), and metal pollution index (MPI) during the different seasons were calculated and presented in Figs. 4 and 5. Based on the average weight of a person and the average daily consumption of fish meat, we calculated the estimated daily intake of each microelement (Supplementary material Figure S3). We used these values to calculate a target hazard quotient, the results of which demonstrated or disproved the potentially harmful effects of consuming such meat. The overall trend for THQ (with no season as a factor) was as follows: Ni > Zn > Hg > Fe > Ba > Cu > Al > Li > Sr > Mn > Cr. Among the seasons, statistically significant higher THQ values for Al, Cr, Fe, and Ni were observed in autumn. Conversely, Ba, Li, and Sr exhibited statistically significant higher THQ values in the summer season. The values of THQ for Al, Cr, and Ni, and THQ for Al, Cr, and Ni, differed significantly between seasons (*p* < 0.001) indicates a lower risk of contamination by these elements during the summer season. With no statistically significant differences between seasons, the higher THQ values for Cu were observed in autumn season. Although no statistically significant differences were observed between seasons, higher THQ values for Hg, Mn, and Zn were found in the summer season. The average annual intake of fish meat for the average European has been set at 24 kg by the European Commission (2019) (Food, Farming, Fisheries, Oceans and Fisheries, Consumption, Directorate-General for Maritime Affairs and Fisheries., 2019). We have therefore calculated the daily intake at an average of 65.75 g of fish meat. All individual THQ values were below 1 (Fig. 4). The total THQ (TTHQ), as the sum of THQs of all heavy metals observed in our study, also showed a value less than 1 for individuals in both seasons. The higher MPI value was calculated in autumn season with an average 1.96, followed by 1.87 in summer season. These results suggest no statistically significant difference between seasons in MPI values (Fig. 5).

## Discussion

This research aims to assess the potential associations between microelement levels and biomarkers such as fatty acids and oxidative stress markers in fish muscle. The selected species, grass carp, is a herbivore farmed worldwide, especially in Asia, making it an important economic species in aquaculture [62, 63].

The main tested toxic elements in fish are typically mercury, cadmium, and lead [64, 65], which is understandable due to their numerous adverse effects on an individual’s health. However, their concentrations are often relatively low compared to essential trace elements (Fe, Cu, or Zn), which can easily become toxic at high concentrations [6, 33]. Previous studies have shown variations in metal content in fish muscle based on factors such as location [66, 67], species [4, 68], age [69], or season [70, 71]. In our study, the concentrations of Cd, and Co were below the limit of quantification in all monitored samples. Concentrations of As, Pb, and Se were detected in a small number of our samples. These values (Cd, Co, and Pb) were several times lower than those detected by Andreji et al. [4] in *Cyprinidae* fish in the area of the Nitra river (about 50 km from our location), which could be caused by long-term pollution of this river, as well as contaminated sediment after an ecological accident in the sixties of the last century [72]. The authors confirmed Pb concentrations from 0.30 to 34.59 mg/kg in all *Cyprinidae* fish muscle samples, which is many times higher than prescribed Commission Regulation (EU) 2023/915 (max. 0.30 mg/kg) [73]. They also found above-limit values for Cd and Hg, and lower Ni content, which is inconsistent with our results; however, Cu, Mn, and Zn content in muscle tissue is comparable with our study. Comparable observations of trace element concentrations have been reported in two *Cyprinidae* freshwater species (*Capoeta tinca* and *Squalius pursakensis*) in Turkey (freshwater reservoir) [10]. Concentrations of lead and cadmium in muscle were not detected, partially comparable to our results. However, the levels of Ba (ranged from 3.74 to 8.03 mg/kg), Cu (ranged from 0.79 to 1.82 mg/kg), Fe (ranged from 15.32 to 57.42 mg/kg), Ni (ranged from 0.47 to 8.52 mg/kg), Sr (ranged from 6.47 to 14.17 mg/kg), or Zn (ranged from 19.19 to 43.72 mg/kg), were several times higher in our muscle samples. Aluminum was the third most represented element (ranged from 9.00 to 38.81 mg/kg) within the monitored element matrix. This metal is not a standard member of the monitored “toxic panel” like mercury, cadmium, or lead, which is not justified. It meets several criteria warranting increased attention, such as an undetermined biological function [74], toxicity at high doses [75], pathologies of internal organs [76, 77], induction of oxidative stress [78], and constant increase of concentrations in the environment due to natural and anthropogenic activities. Liu et al. [79] studied tissues of grass carp from various locations around a copper mine, where they did not detect cadmium. The concentrations of Cr, Hg, Mn, Ni, and Zn were lower (0.052 ± 0.025 μg/g w. w., for Cr, 0.006 ± 0.002 μg/g w. w., for Hg, 0.32 ± 0.16 μg/g w. w., for Mn, 0.015 ± 0.009 μg/g w. w., for Ni, and 4.87 ± 0.67 μg/g w. w. for Zn) in muscle tissue than our findings. However, they also confirmed the presence of lead (0.128 ± 0.042 μg/g w. w.) and higher values of copper in samples (1.60 ± 0.18 μg/g w. w.).

Seasonal variations for Fe and Zn in common carp muscle were confirmed by Tekin-Özan and Kir [80]. Over 2 years, they observed a higher Fe content in autumn compared to summer, consistent with our findings. The Cu content was balanced in the first year but showed a lower concentration in autumn during the second year of testing, possibly associated with fish activity. The authors did not detect Cu or Mn in the muscle samples. Wang et al. [81] evaluated seasonal variations of heavy metals in crucian carp muscle. They found several significant differences, generally with the highest levels of metals (Cd, Cr, Cu, Mn, Ni, Pb, and Zn) occurring in the winter season. However, comparable tendencies to ours were found for Cr concentrations, with a significant increase observed in autumn compared to the summer season. Conversely, the concentrations of Zn, Pb, Cu, Mn, and Ni showed opposite trends. As can bioaccumulate in fish by several processes, but dietary intake is probably the primary route [82]. This can directly affect the seasonality. For instance, the food intake of grass carp decreases with decreasing water temperature [83], which could have caused the non-detection of As in the autumn season.

The fatty acid composition in fish refers to the types and proportions of fatty acids present in the lipids of fish tissues. In Sun et al. [84] study, the muscle of adult grass carp showed the highest levels of monounsaturated fatty acids (MUFA), followed by saturated fatty acids (SFA) and polyunsaturated fatty acids (PUFA). However, our present study contradicts these findings, as the muscle SFA content was more represented than MUFA content, which may be caused by a different ecological environment [85]. Seasonal changes in FAs were detected. An important finding is the significantly higher proportions of EPA + DHA in summer, confirming the higher nutritional value of fish during this season. Guler et al. [86] confirmed a significantly higher representation of DHA in the muscle of common carp during the summer season compared to all other seasons. EPA had a higher ratio in summer than in autumn, but lower than in spring or winter. The significantly higher nutritional value and proportional composition of fatty acids associated with the summer season could be attributed to increased water temperature, food abundance, and thus increased intake [87]. Das et al. [29] investigation indicates that alterations in the content of polyunsaturated fatty acids (PUFA), monounsaturated fatty acids (MUFA), and omega fatty acids, following the accumulation of heavy metals (Pb, Cu, Cd, Ni, and Zn), can lead to significant oxidative damage and subsequent mitochondrial dysfunction. The study suggests that an increase in enzymatic antioxidant activity is an effort to sustain redox balance and counterbalance changes in the muscle fatty acid profile. Hg can affect the levels of EPA+DHA in fish muscle, which may impair the nutritional quality and health benefits of fish for human consumption; however, it is mainly related with water chemistry [88]. Hg and Al can induce oxidative stress and damage the tissue of fish [40, 78], which may affect the quality and safety of fish for human consumption. Therefore, it is important to monitor the levels of Hg and Al in fish muscle and limit the exposure of fish to these metals. The positive association between Al and SOD levels, and between Hg and Ba with GPx levels, may indicate the previously described role of enzymatic antioxidants as the first line of defence against oxidative stress [89, 90]. Ba generally does not raise significant concerns regarding fish toxicity; minimal risks have been described [91]. However, there are also indications of toxic effects for other freshwater biota [92]. We described the associations of Ba with several biochemical parameters of grass carp blood in our previous study [42], e.g., the effect on the levels of creatinine, uric acid, glucose, AST, TP, or the mineral profile (Na, K, and P). Sr, commonly found in the environment, accumulates relatively well in fish and is generally not considered a problem [93]. However, when compared to toxic elements (Cd, Pb, or Hg), it was Sr that influenced the spinal deformities of cod [94], association with calcium. The interference of Sr with calcium or zinc ions can subsequently affect enzymes or regulatory proteins, potentially leading to oxidative imbalance [90].

The surpassing of established limits for toxic metals as stipulated by various regulatory bodies does not consistently signify a strict risk to human health. As a result, in recent years, there has been widespread utilization of health risk assessment methods to appraise the influence of the bioaccumulation of heavy metals on human health hazards [37]. By employing the THQ or TTHQ, we analyze the quantity of a specific substance that an individual may encounter daily from different sources, such as fish meat. The objective is to determine whether the calculated intake remains within acceptable limits or if it presents potential health risks. All THQ values were also below 1, indicating that consumption of these fish is safe with no health risks. The TTHQ as the sum of THQs of all heavy metals observed in our study also showed a value less than 1 for individuals in all three seasons; hence, we consider the consumption of these fish to be safe. MPI values ranged from 1.42 to 2.54 on average in the monitored groups; the overall average was 1.91. As our results show, metal pollution index is low, and there should be no risk of high contamination of heavy metals in fish muscle. Hossain et al. [95] evaluated heavy and trace metals (Pb, Cr, Cu, Zn, Mn, Fe, Hg, Ni, Ca, Co, Se, Rb, Sr, and As) bioaccumulation in 15 species of fish from different feeding habitats in Bangladesh. For our comparison, the findings for *Ctenopharyngodon idella*, *Labeo rohita*, and *Aristichthys nobilis* (family *Cyprinidae*) are interesting. The results of the hazardous index (HI) values were below 1, indicating the safe consumption of fish from these locations, despite MPI values being several times higher than in our study (13.24 for *C. idella*, 16.83 for *L. rohita*, and 13.25 for *A. nobilis*). Habib et al. [11] studied metal concentration in organs and muscle of *Labeo rohita* (*Cyprinidae* family). They found that the THQ values were below 1 for Cu, Cd, Pb, and Zn. However, the THQ for Cr (> 1) indicated a potential health risk for consumers. The overall hazardous index did not exceed the value of 1, but wild fish HI was higher than farm fish. In general, it was found that the main components forming TTHQ in our study are Ni, Zn, and Hg. Although all THQ values were below 1, Ni and Hg deserve particular attention as they together constitute 49% of the TTHQ. Additionally, it is essential to consider other elements, especially due to the potential synergistic effect [12], which could pose a threat to more vulnerable population groups, such as children and the elderly.

Today, food safety is a priority of almost every country in the world, as well as many organizations such as the United Nations or the European Union. The findings from this research partially align with prior data on the *Cyprinidae* family, indicating lower levels of polyunsaturated fatty acids (PUFA) and higher concentrations of saturated fatty acids (SFA) and monounsaturated fatty acids (MUFA), along with elevated levels of DHA vs. EPA [29, 84]. Despite the proven health benefits of fatty acids, it is still important to consider the presence of pollutants even in varying amounts. Differences between trace elements and observed oxidative stress markers or FAs in fish serve as effective indicators for assessing exposure to metals. Oxidative stress has the potential to induce lipid peroxidation and deplete enzymatic antioxidants in muscles. Encouragingly, the discovery of low values (up to 1) in THQ and TTHQ is comparable with the authors’ earlier research. Furthermore, MPI values in the current study suggest low environmental pollution.

## Conclusions

This study reveals various associations between specific trace elements found in the tissues and several indicators such as SOD, GPx, TAS levels, or FAs composition. Notably, the concentration of the typical toxicant (Cd) was consistently below the limit of quantification (< LOQ) in all grass carp muscle samples. Additionally, the concentration of Pb, as the second toxic element, was lower than the LOQ in more than 66% of the samples. Fe concentration was observed to be the highest (15.32–57.42 mg/kg), whereas Hg levels were the lowest (0.012–0.053 mg/kg). The levels of Ba, Li, and Sr were significantly higher in the summer season, contrary to the significantly higher concentrations of Al, Cr, Fe, and Ni in the autumn season. Seasonal variations were evident in the fatty acid composition and levels of glutathione peroxidase (GPx) as an enzymatic antioxidant. The proportional composition of FAs defined significantly higher EPA and DHA as the main nutritional components in the summer season. The study employed correlations, principal component analysis (PCA), and cluster analysis, uncovering numerous associations between the monitored elements and biomarkers. The health risk assessment indices, including target hazard quotient (THQ), total target hazard quotient (TTHQ), and metal pollution index (MPI), collectively indicate low contamination (THQ and TTHQ below 1; MPI values ranging from 1.42 to 2.54) of both non-essential and essential elements. Importantly, low risks associated with the consumption of grass carp from the tested location were identified.

## Supplementary Information

ESM 1 (DOCX 319 kb)

## Data Availability

The authors declare that the data support the findings of this study are available from the corresponding author on reasonable request.
