# Supplementary material for: Microelements, Fatty Acid Profile, and Selected Biomarkers in Grass Carp (Ctenopharyngodon idella) Muscle Tissue: Seasonal Variations and Health Risk Assessment
Source: Biol Trace Elem Res. 2024 May 9;203(2):1048–63. doi: 10.1007/s12011-024-04190-9 (PMC11750936; doi:10.1007/s12011-024-04190-9)
Supplement: Supplementary file 1 — (DOCX 319 kb) [file 12011_2024_4190_MOESM1_ESM.docx]

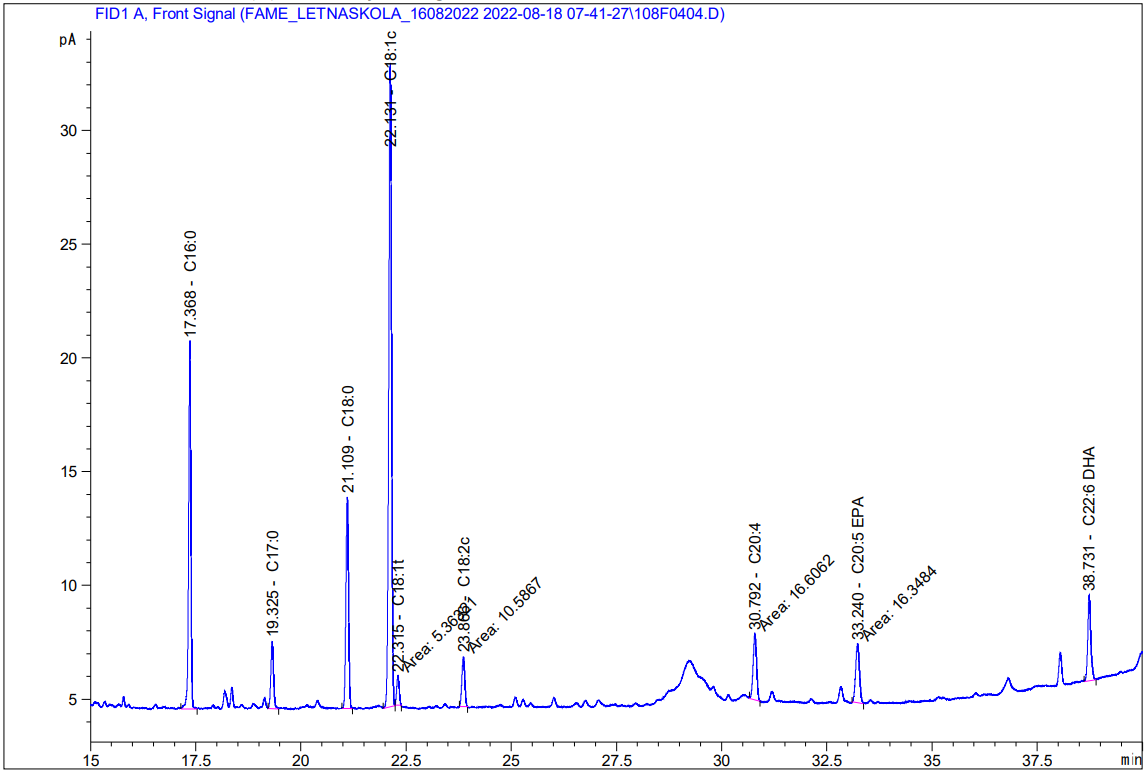
**Figure S1.** GC chromatogram of fish sample analysis


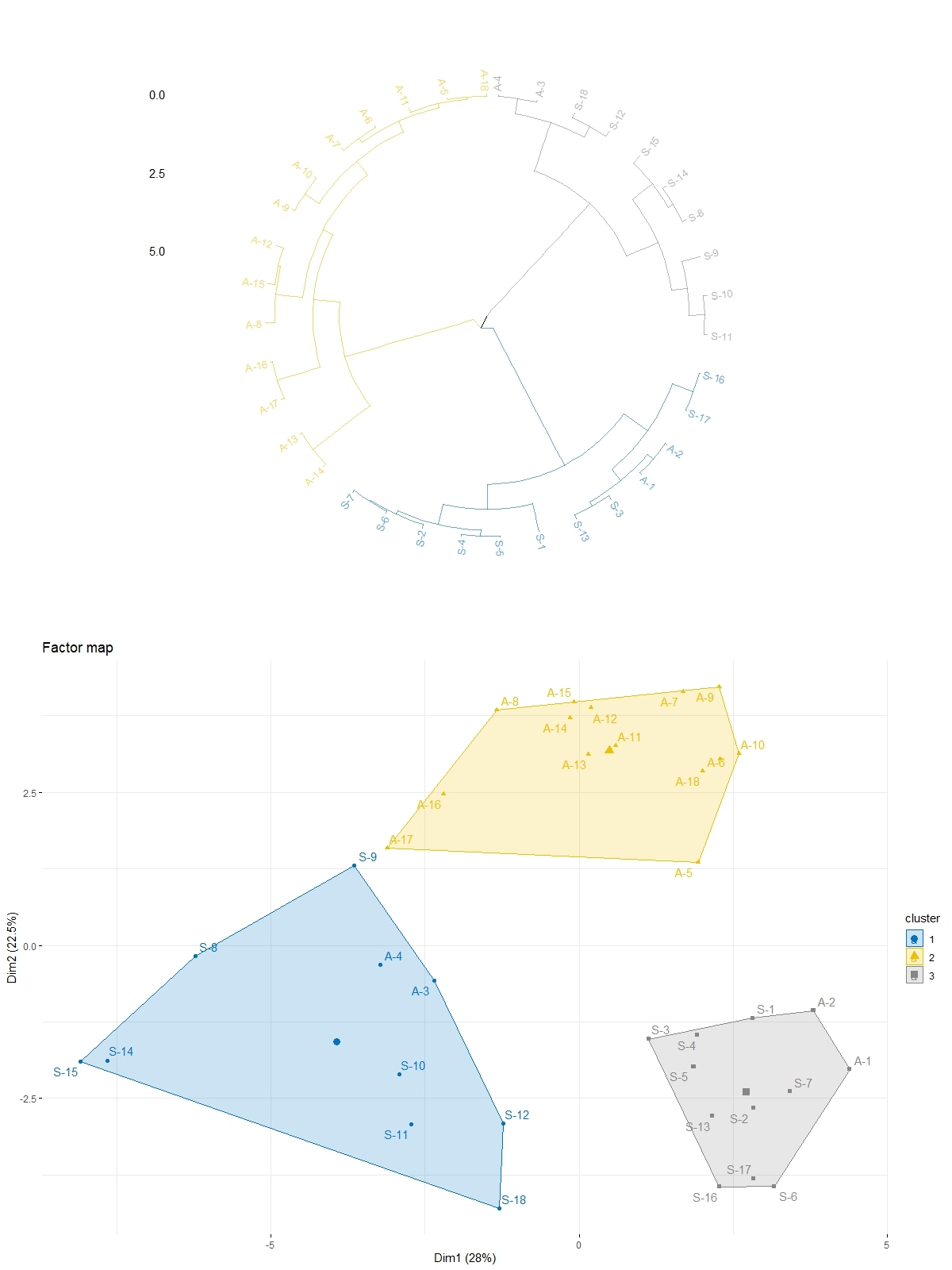


**Figure S2** Cluster dendrogram and Factor map.

**Figure S3** Results of estimated daily intake (EDI) (µg/kg/person); columns represent mean ± SD; the level of significance was set at **p* < 0.05; ** *p* < 0.01; *** *p* < 0.001.
